# Synthesis and pharmacological evaluation of mono-arylimidamides as antileishmanial agents

**DOI:** 10.1016/j.bmcl.2016.03.082

**Published:** 2016-05-15

**Authors:** Xiaohua Zhu, Abdelbasset A. Farahat, Meena Mattamana, April Joice, Trupti Pandharkar, Elizabeth Holt, Moloy Banerjee, Jamie L. Gragg, Laixing Hu, Arvind Kumar, Sihyung Yang, Michael Zhuo Wang, David W. Boykin, Karl A. Werbovetz

**Affiliations:** a Division of Medicinal Chemistry and Pharmacognosy, College of Pharmacy, The Ohio State University, Columbus, OH 43210, USA; b Department of Chemistry, Georgia State University, Atlanta, GA 30302, USA; c Department of Pharmaceutical Organic Chemistry, Faculty of Pharmacy, Mansoura University, Mansoura 35516, Egypt; d Institute of Medicinal Biotechnology, Chinese Academy of Medical Sciences and Peking Union Medical College, Beijing 100050, China; e Department of Pharmaceutical Chemistry, School of Pharmacy, University of Kansas, Lawrence, KS 66047, USA

**Keywords:** Leishmaniasis, Arylimidamide, DB766, Drug discovery

## Abstract

Arylimidamide (AIA) compounds containing two pyridylimidamide terminal groups (bis-AIAs) possess outstanding in vitro antileishmanial activity, and the frontrunner bis-AIA DB766 (2,5-bis[2-(2-isopropoxy)-4-(2-pyridylimino)aminophenyl]furan) is active in visceral leishmaniasis models when given orally. Eighteen compounds containing a single pyridylimidamide terminal group (mono-AIAs) were synthesized and evaluated for their antileishmanial potential. Six of these compounds exhibited sub-micromolar potency against both intracellular *Leishmania donovani* and *Leishmania amazonensis* amastigotes, and three of these compounds also displayed selectivity indexes of 25 or greater for the parasites compared to a J774 macrophage cell line. When given orally at a dose of 100 mg/kg/day for five days, compound **1b** (*N*-(3-isopropoxy-4-(5-phenylfuran-2-yl)phenyl)picolinimidamide methanesulfonate) reduced liver parasitemia by 46% in *L. donovani*-infected mice. Mono-AIAs are thus a new class of candidate molecules for antileishmanial drug development.

## Graphical abstract

**Figure:**
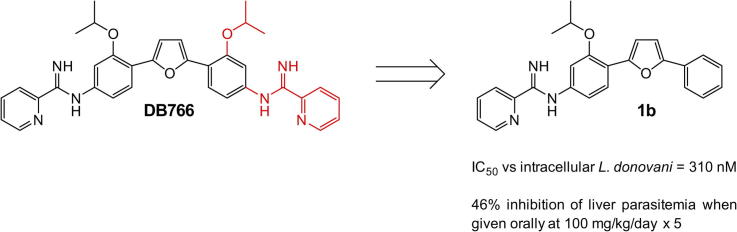

With well over one million new cases and 20,000–40,000 deaths annually,^1^ leishmaniasis is the second most prevalent infectious disease transmitted by a vector in terms of morbidity and mortality.^2^ Leishmaniasis is a spectrum of disease caused by infection with approximately twenty species of *Leishmania* protozoa. Among the major clinical forms, visceral leishmaniasis (VL) is the most severe and is fatal if left untreated, while cutaneous leishmaniasis (CL) is typically self-healing. Nevertheless, drugs are frequently used to treat CL to limit scarring from cutaneous lesions, to speed the time to cure, and to prevent the development of the potentially disfiguring mucocutaneous leishmaniasis manifestation. The current drugs against leishmaniasis include pentavalent antimonials, amphotericin B (AmB), miltefosine and paromomycin, but they all suffer from one or more weaknesses, such as parasite resistance, toxicity, high cost, and an inconvenient route of administration.^2^ New oral antileishmanial drugs are thus needed to overcome these issues and provide better treatment options against *Leishmania* infections.

We have demonstrated that bis-arylimidamides (bis-AIAs), a series of compounds containing two pyridylimidamide terminal groups, possess excellent in vitro and promising in vivo antileishmanial activity. In the AIAs, the imino group is bound to an anilino nitrogen atom, lowering the p*K*_a_ of the amidine and increasing the lipophilicity of the molecule compared to a dicationic diamidine compound such as pentamidine.^3^ DB766 and DB1960, the hydrochloride and mesylate salts of 2,5-bis[2-(2-isopropoxy)-4-(2-pyridylimino)aminophenyl]furan, respectively, display IC_50_ values similar to AmB against both intracellular *Leishmania donovani* and intracellular *Leishmania amazonensis* and provide dose-dependent reduction of liver parasitemia in a mouse model of visceral leishmaniasis when administered orally.^3^, ^4^ Unfortunately, DB766 and DB1960 lack the therapeutic window required for advancement as clinical monotherapy candidates against visceral leishmaniasis.^4^

As part of a broader study, we previously reported two terphenyl AIAs containing a single pyridylimidamide group (mono-AIAs) that displayed promising activity against intracellular *L. amazonensis*.^5^ We hypothesize that lower molecular weight mono-AIAs may have improved pharmacokinetic properties and could display decreased toxicity to animals compared to bis-AIAs. The synthesis and evaluation of such a group of molecules is reported here to test this hypothesis.

Most of the mono-AIAs investigated were synthesized as outlined in Scheme 1 using methodology similar to that employed previously for making bis-AIAs.^6^, ^7^, ^8^ The first step involved Stille coupling between tributyl(furan-2-yl)stannane and various substituted 4-bromonitrobenzenes. Bromination of the 2-(4-nitrophenyl)furan analogs with NBS at room temperature yielded the corresponding 5-bromo-2-(4-nitrophenyl)furans. Subsequent reaction of the 5-bromofurans under either Stille or Suzuki conditions provided the 2-(4-nitrophenyl)-5-arylfuran intermediates. Catalytic hydrogenation of the nitro group of the diarylfurans gave the corresponding amino analogs. Finally, reaction of the amino diarylfurans with either 2-pyridyl or 2-pyrimidyl thioimidate analogs produced, after salt formation, the mono-AIAs **1a**–**p**. Most target compounds were prepared as the more soluble mesylate salt.^4^ Hydrochloride salts were made for **1a** and **1d** because of the hygroscopic nature of the corresponding mesylates.

The synthesis of triazole **2** employed the Huisgen cycloaddition process under typical ‘Click chemistry’ conditions to yield the key nitro intermediate 4-(2-isopropoxy-4-nitrophenyl)-1-(4-methoxyphenyl)-1*H*-1,2,3-triazole as shown in Scheme 2.^9^ The subsequent steps are similar to those of Scheme 1 but the reduction of the nitro group to the amine in this case was achieved using Raney-nickel and hydrazine.^10^

The synthesis of the oxazole **3** is presented in Scheme 3 and employed (*p*-toluenesulfonyl)methyl isocyanide chemistry for the formation of 5-(2-isopropoxy-4-nitrophenyl)oxazole.^11^ The latter compound was converted into 2-phenyl-5-(2-isopropoxy-4-nitrophenyl)oxazole by Pd/Cu catalyzed arylation with iodobenzene.^12^, ^13^ The conversion of the nitro analog into the mono-AIA **3** followed methodology previously described in Scheme 1.

Methods employed for the evaluation of the efficacy of mono-AIA target compounds against intracellular *L. amazonensis*^14^ and intracellular *L. donovani*^15^ and for toxicity to murine J774 macrophages^16^ have been described previously. In these assays, the reference compound amphotericin B displayed IC_50_ values of 40 ± 3 nM (mean ± standard error, *n* = 15) and 120 ± 10 nM (mean ± standard error, *n* = 26) against *L. donovani* and *L. amazonensis*, respectively, while the cytotoxicity standard podophyllotoxin showed an IC_50_ value of 24 ± 5 nM (mean ± standard error, *n* = 15) against J774 macrophages. For ease in illustrating the antileishmanial SAR of this series of compounds, the central phenyl ring, the linker, and the terminal phenyl ring are referred to as rings A, B, and C, respectively (Fig. 1).

For derivatives bearing substitutions at the 5 position of the furan ‘B’ ring (at the position occupied by the C ring in Fig. 1), we observed that the most active compound was **1b**, where the C ring is a phenyl group. Compound **1b** displayed IC_50_ values of 310 and 130 nM against intracellular *L. donovani* and intracellular *L. amazonensis*, respectively (Table 1). Activity was maintained when the phenyl ring was replaced with a furan ring (**1d**). The antileishmanial potency decreased by 4–20 fold when this position was substituted with other groups, however, such as hydrogen (**1a**), 5-pyrimidyl (**1c**), 3-pyridyl (**1e**), or 4-pyridyl (**1f**).

The addition of a methoxy group or a fluorine atom at position R^2^ or a methoxy group at position R^1^ of phenyl ring C led to decreased antileishmanial potency in both the intracellular *L. amazonensis* and *L. donovani* assays (Table 2). In the series of compounds bearing different substitutions on phenyl ring ‘A’, the cyclopentyloxy substituted derivative **1k** was the most active compound, with IC_50_ values comparable to that of **1b** and amphotericin B (Table 3). As the size of the alkoxy substituent increased from methoxy (**1m**) to ethoxy (**1l**) to isopropoxy (**1b**) and cyclopentyloxy (**1k**), antileishmanial activity increased, but switching the position of the isopropoxy substitution on the ‘A’ ring (**1j**) resulted in a loss of antiparasitic activity. Replacement of furan as the ‘B’ ring with other heterocycles (thiophene **1o**, triazole **2**, or oxazole **3**) lowered the antileishmanial activity, with **3** being intermediate in potency between the highly active **1b** and the moderately active **2** and **1o** (Table 4). Replacement of the 2-pyridyl terminal group with a 2-pyrimidyl group (**1p**) resulted in a reduction in potency against *L. amazonensis* but not against *L. donovani* (Table 4). In the host cell counterscreen, the mono-AIAs exhibited IC_50_ values ranging from 5300 to >50,000 nM against murine J774 macrophages, resulting in selectivity indexes (IC_50_ vs J774 macrophages/IC_50_ vs *Leishmania*) of 2.4–100 against intracellular *L. amazonensis* and 3.1–76 against intracellular *L. donovani* in vitro.

Among these 18 mono-AIAs, **1b**, **1d**, and **1k** displayed outstanding antileishmanial potency and good selectivity for intracellular *Leishmania* compared to J774 murine macrophages (selectivity indexes ⩾25), warranting the in vivo evaluation of these derivatives. Compounds **1b**, **1d**, and **1k** were dissolved in water and administered to healthy BALB/c mice for assessment of their in vivo toxicity. Each of these compounds was well tolerated when administered by the i.p. route at 30 mg/kg/day for 5 days and were thus evaluated at this dose in a murine model of visceral leishmaniasis.^4^ Animals were infected with *L. donovani* LV82 promastigotes and then treated with different compounds one week post infection for five consecutive days. These mice were euthanized two weeks post infection and liver smear slides were prepared for the microscopic determination of parasitemia. When given at the dose listed above, administration of **1b**, **1d**, and **1k** resulted in 37%, 13%, and 20% suppression of liver parasitemia, respectively, compared to untreated control groups (Fig.2A, B). As the most effective of these three compounds when given i.p., the oral efficacy of **1b** was also evaluated in the murine visceral leishmaniasis model. Compound **1b** reduced liver parasitemia by 46% at an oral dose of 100 mg/kg/day for five days compared to the control group (Fig.2C). The in vivo antileishmanial efficacy of **1b** is thus similar to that of DB1960 and lower than that of DB766 when the compounds are administered orally at 100 mg/kg in five daily doses.^3^, ^4^ When given to infected mice at a dose of 10 mg/kg/day for five days by either the i.p. or oral route, miltefosine administration resulted in >90% inhibition of liver parasitemia, consistent with our previous observations.^4^, ^15^

To confirm the exposure and accumulation of **1b** in target organs after oral administration, plasma and tissue concentrations of this compound were determined at 1, 2 and 24 h after a single oral dose at 100 μmol/kg (or 40 mg/kg) in mice. Compound **1b** accumulated in the target organs such as liver and spleen at higher concentrations (C1h = 12.6 and 4.7 μM, respectively) than in the plasma (C1h = 1.9 μM) (Fig. 3). At 24 h post dose, **1b** concentrations decreased substantially to 0.14 μM in the plasma and 0.6–0.7 μM in the liver and spleen.

Our efforts to explore the mechanism of antileishmanial action of the bis-AIAs led to the observation that DB766 is synergistic with posaconazole against *Leishmania donovani* in vitro^17^; other investigators reported in vitro antileishmanial synergy between posaconazole and the squalene synthase inhibitor E5700.^18^ A combination study with **1b** and posaconazole was thus performed against intracellular *L. amazonensis* amastigotes. While azoles have been used to treat CL,^19^, ^20^ some of these compounds exhibit low potency against certain *Leishmania* species in vitro.^21^, ^22^ We found this to be true for posaconazole against intracellular *L. amazonensis* under the conditions of our assay (IC_50_ > 10 μM; posaconazole exhibits low solubility at higher concentrations), making standard isobologram analysis impossible in this case. Instead, the method described by Peters et al.^23^ was employed to determine whether posaconazole enhances the potency of **1b**. Figure 4 demonstrates that this azole augmented the potency of the mono AIA **1b** against intracellular *L. amazonensis*, with **1b** displaying an IC_50_ value that is fivefold lower in the presence of 8 μM posaconazole compared to the IC_50_ of this AIA in the absence of the azole. IC_50_ values for **1b** (DB2002) alone in this experiment and in the presence of 8 μM posaconazole are 0.26 ± 0.05 and 0.055 ± 0.015 μM, respectively. We are aware that de Macedo-Silva et al. reported an IC_50_ value of 1.6 μM for posaconazole against intracellular *L. amazonensis*.^24^ Although both assays involved incubating infected murine peritoneal macrophages with posaconazole for 72 h, de Macedo-Silva exposed peritoneal macrophages to *L. amazonensis* for a shorter time compared to our studies prior to removing extracellular parasites (2 h vs overnight). Our studies with intracellular *L. donovani* have indicated that the level of macrophage infection influences the apparent in vitro antileishmanial activity of azoles (A. Joice, unpublished data). Thus, a likely explanation for the discrepancy between IC_50_ values for posaconazole against intracellular *L. amazonensis* in the two studies is the differences in assay conditions employed.

In summary, eighteen mono-AIAs were synthesized and evaluated for their antileishmanial activities. Compounds **1b**, **1d** and **1k** displayed the highest potency against intracellular *L. amazonensis* and intracellular *L. donovani* amastigotes with their IC_50_ values slightly higher than those of amphotericin B and DB766. These three mono-AIAs were well tolerated in healthy mice, indicating that mono-AIAs may be generally less toxic than bis-AIAs.^8^, ^25^ Compound **1b** shows moderate in vivo antileishmanial efficacy in a mouse model of visceral leishmaniasis. Furthermore, posaconazole enhances the activity of this compound against intracellular *L. amazonensis* amastigotes, consistent with our observations with the bis-AIA DB766.^17^ Mono-AIAs are thus promising antileishmanial leads, and further optimization of this class of compounds to improve their in vivo efficacy is needed. Given the in vitro activity of the compound **1b**/posaconazole combination, combining a mono AIA with an azole drug could be a fruitful strategy for the development of antileishmanial therapies.

## Figures and Tables

**Figure 1:**
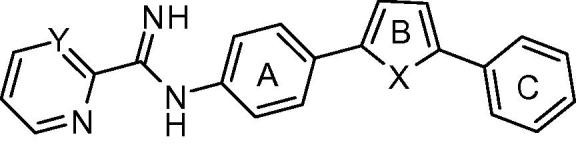
Mono-AIA scaffold.

**Figure 2:**
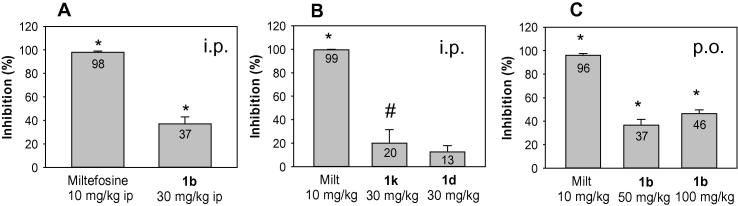
In vivo efficacy of mono-AIAs in the murine visceral leishmaniasis model. Compounds were administered i.p. (A and B) or p.o. (C) once daily for 5 days to infected mice. Results are presented as the percentage inhibition of liver parasitemia. Bars and error bars show the mean and standard deviation, respectively (*n* = 4) ^*^p < 0.01, compared to untreated control, ^#^*p* < 0.05, compared to untreated control.

**Figure 3:**
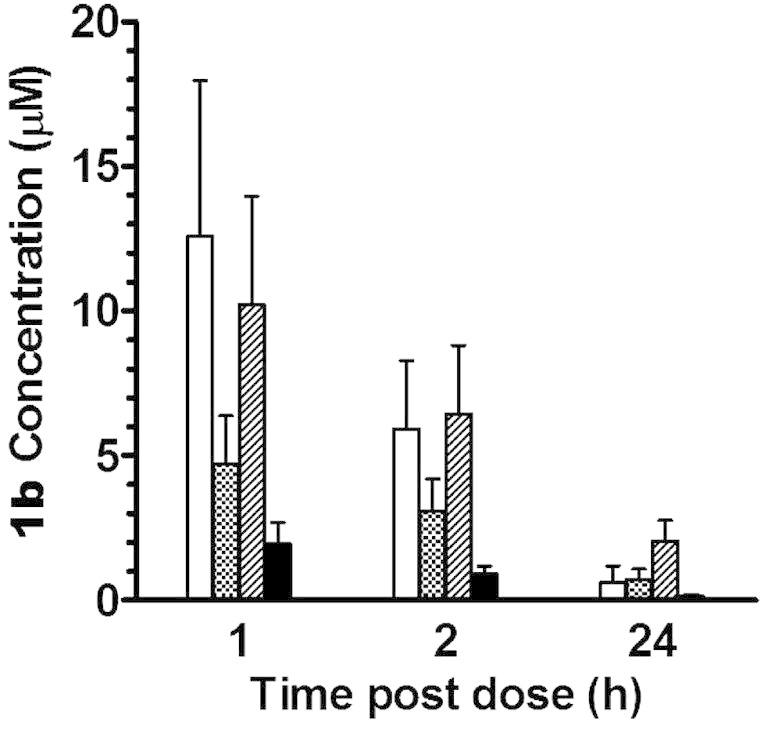
Concentration of **1b** in liver (), spleen (), kidney (), and plasma () after oral administration at a dose of 100 μmol/kg (or 40 mg/kg) to mice. Bars and error bars represent means and standard errors of triplicate determinations, respectively.

**Figure 4:**
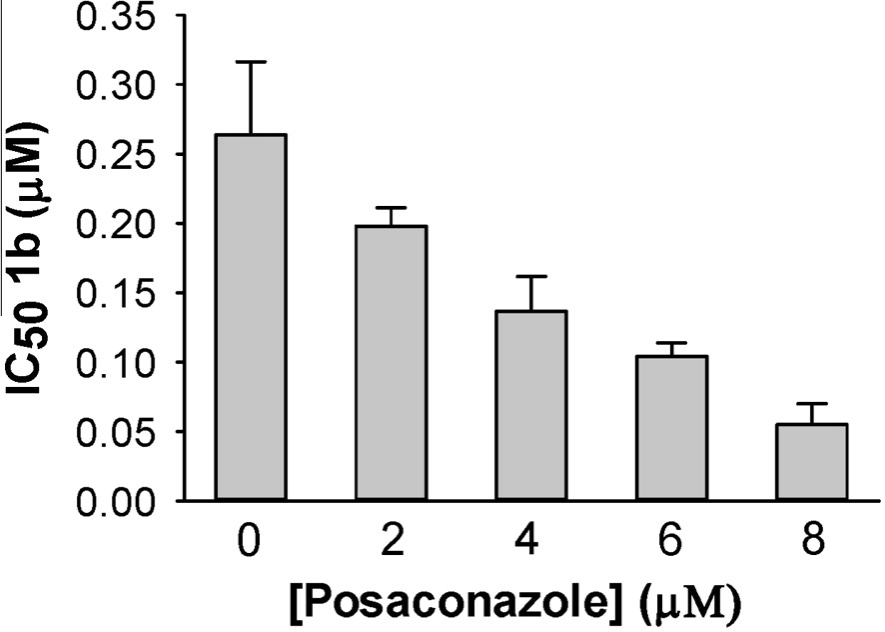
In vitro interactions between **1b** and posaconazole against intracellular *L. amazonensis*. Results are presented as the IC_50_ of **1b** alone and in different combinations. Bars and error bars show the mean and standard deviation of three independent experiments.

**Scheme 1:**
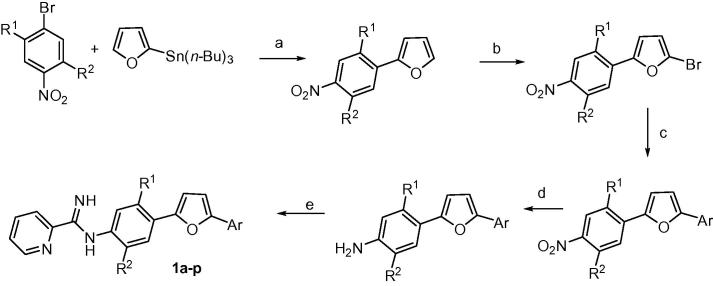
Reagents and conditions: (a) Pd(PPh_3_)_4_, 1,4-dioxane, reflux, 68–79%; (b) NBS, DMF, rt, 72–85%; (c) ArSn(*n*-Bu)_3_, Pd(PPh_3_)_4_, 1,4-dioxane, reflux or ArB(OH)_2_, Pd(PPh_3_)_4_, toluene, Na_2_CO_3_, H_2_O, 80 °C, 59–79%; (d) H_2_, Pd/C, EtOAc, EtOH, 68–98%; (e) (i) *S*-(2-naphthylmethyl)-2-pyridylthioimidate hydrobromide, EtOH/CH_3_CN, rt, (ii) HCI/EtOH or CH_3_SO_3_H, CH_2_CI_2_, 55–73%.

**Scheme 2:**
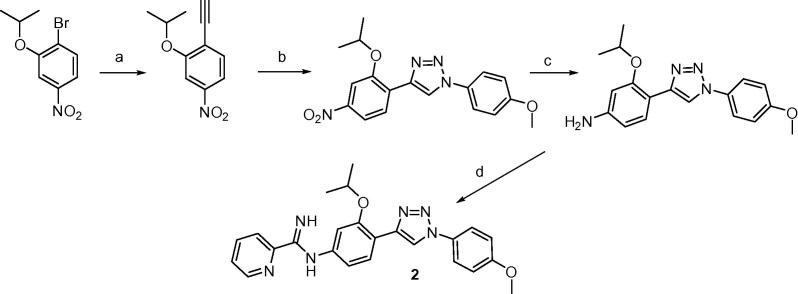
Reagents and conditions: (a) (i) ethynyltrimethylsilane, PdCI_2_(PPh_3_)_2_/Cul,PPh_3_/Et_3_N, 85%, (ii) K_2_CO_3_, MeOH, 94%; (b) 4-methoxyphenylazide, CuSO_4_-5H_2_O, l-ascorbate, *t*-BuOH, H_2_O, 38%; (c) Ra-Ni, NH_2_NH_2_, MeOH, 50 °C, 89%; (d) (i) *S*-(2-naphthylmethyl)-2-pyridylthioimidate, EtOH, CH_3_CN, rt, (ii) CH_3_SO_3_H, CH_2_CI_2_, 61%.

**Scheme 3:**
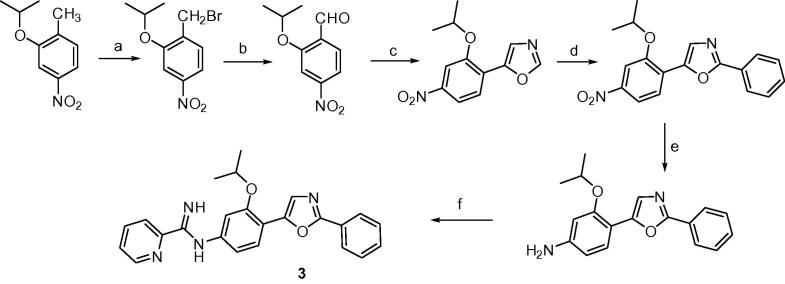
Reagents and conditions: (a) NBS, AIBN, CCI_4._ light, 89%; (b) Ag_2_O, pyridine N-oxide, MeCN, 81%; (c) toluenesulfonylmethyl isocyanide, K_2_CO_3_, MeOH, 73%; (d) iodobenzene, Pd(OAc)_2_, Cs_2_CO_3_, Cul, DMF, 160 °C, 95%; (e) Ra-Ni, NH_2_NH_2_, MeOH, 50 °C, 94%; (f) (i) *S*-(2-naphthylmethyl)-2-pyridylthioimidate hydrobromide, EtOH/CH_3_CN, rt, (ii) CH_3_SO_3_H, CH_2_CI_2_, 71%.

**Table 1:** In vitro activity of compounds (**1a**–**f**) against intracellular *Leishmania* amastigotes and murine J774 macrophages

**Figure:**
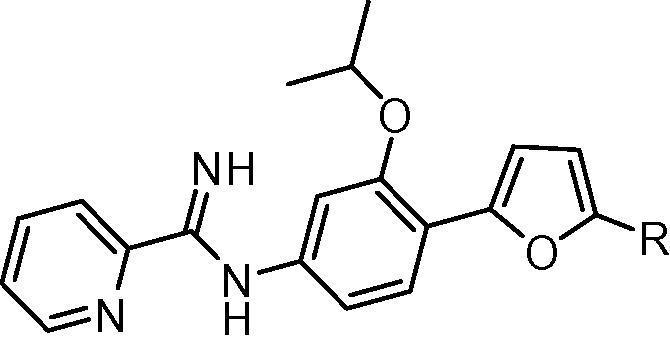

Compound	R	IC_50_^a^ (nM)		
Intracellular *L. amazonensis*	Intracellular *L. donovani*	J774 macrophages
**1a**	H	2100 ± 200	2400 ± 300	22,000 ± 4000
**1b**		130 ± 30	310 ± 10	13,000 ± 1000
**1c**		1100 ± 300	2100 ± 400	>20,000
**1d**		250 ± 10	340 ± 50	8500 ± 800
**1e**		900 ± 230^b^	1200 ± 100	>50,000
**1f**		2700 ± 200^b^	2600 ± 100	22,000 ± 1000

a The values are means ± standard errors of at least three independent experiments.

b The values are the means ± range of two independent experiments.

**Table 2:** In vitro activity of compounds **1b**, **1g**–**i** against intracellular *Leishmania* amastigotes and murine J774 macrophages

**Figure:**
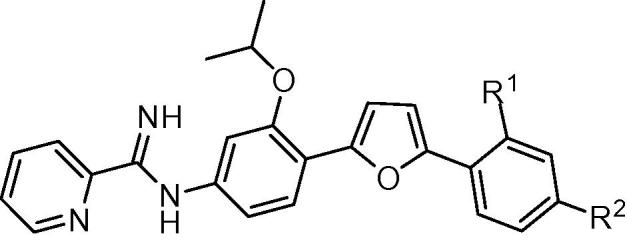

Compound	R^1^	R^2^	IC_50_^a^ (nM)		
Intracellular *L. amazonensis*	Intracellular *L. donovani*	J774 macrophages
**1b**	H	H	130 ± 30	310 ± 10	13,000 ± 1000
**1g**	H	OCH_3_	820 ± 310	900 ± 150	6700 ± 900
**1h**	H	F	920 ± 190	1100 ± 100	8000 ± 100
**1i**	OCH_3_	H	1400 ± 200	1300 ± 100	5300 ± 1000

a The values are means ± standard errors of at least three independent experiments.

**Table 3:** In vitro activity of compounds **1b**, **1j**–**n** against intracellular *Leishmania* amastigotes and murine J774 macrophages

**Figure:**
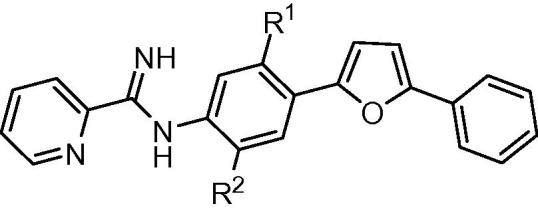

Compound	R^1^	R^2^	IC_50_^a^ (nM)		
Intracellular *L. amazonensis*	Intracellular *L. donovani*	J774 macrophages
**1b**	OCH(CH_3_)_2_	H	130 ± 30	310 ± 10	13,000 ± 1000
**1j**	H	OCH(CH_3_)_2_	1600 ± 300	1400 ± 300	13,000 ± 1000
**1k**	OCH(CH_2_)_4_	H	140 ± 10	170 ± 40	13,000 ± 3000
**1l**	OCH_2_CH_3_	H	980 ± 70^b^	850 ± 170	6600 ± 600
**1m**	OCH_3_	H	3700 ± 1300^b^	1400 ± 300	9000 ± 2000
**1n**	CF_3_	H	>10,000^b^	6100 ± 1400	>20,000

a The values are means ± standard errors of at least three independent experiments.

b The values are the means ± range of two independent experiments.

**Table 4:** In vitro activities of compounds **1b**, **1o**, **1p**, **2**, and **3** against intracellular *Leishmania* amastigotes and murine J774 macrophages

**Figure:**
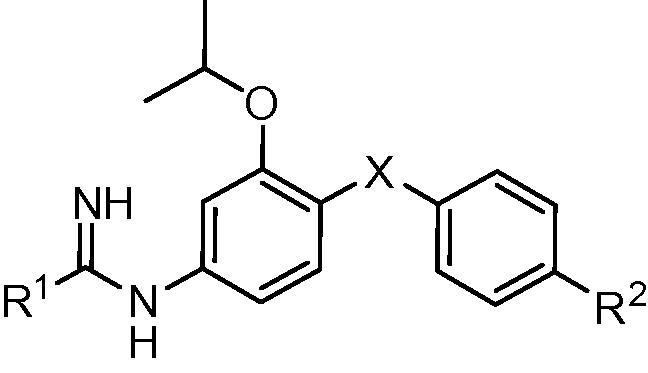

Compound	X	R^1^	R^2^	IC_50_^a^ (nM)		
Intracellular *L. amazonensis*	Intracellular *L. donovani*	J774 macrophages
**1b**		2-Pyridyl	H	130 ± 30	310 ± 10	13,000 ± 1000
**1o**		2-Pyridyl	H	2300 ± 300^b^	2200 ± 500	6900 ± 400
**1p**		2-Pyrimidyl	H	650 ± 160	440 ± 60	12,000 ± 1000
**2**		2-Pyridyl	OCH_3_	2900 ± 100	1600 ± 200	16,000 ± 3000
**3**		2-Pyridyl	H	590 ± 60	1000 ± 0	24,000 ± 1000

a The values are means ± standard errors of at least three independent experiments.

b The value is the mean ± range of two independent experiments.

## Supplementary data

Supplementary data Complete characterizations of target compounds **1b**, **1d**, **1k**, **2**, and **3**.

## References

[1] Alvar J., Vélez I., Bern C., Herrero M., Desjeux P., Cano J., Jannin J., den Boer M. (2012). PLoS One.

[2] Jain K., Jain N. (2013). Drug Discovery Today.

[3] Wang M., Zhu X., Srivastava A., Liu Q., Sweat J., Pandharkar T., Stephens C., Riccio E., Mandal S., Madhubala R., Tidwell R., Wilson W., Boykin D., Hall J., Kyle D., Werbovetz K. (2010). Antimicrob. Agents Chemother..

[4] Zhu X., Liu Q., Yang S., Parman T., Green C., Mirsalis J., Soeiro M., de Souza E., da Silva C., Batista D., Stephens C., Banerjee M., Farahat A., Munde M., Wilson W., Boykin D., Wang M., Werbovetz K. (2012). Antimicrob. Agents Chemother..

[5] Patrick D., Ismail M., Arafa R., Wenzler T., Zhu X., Pandharkar T., Jones S., Werbovetz K., Brun R., Boykin D., Tidwell R. (2013). J. Med. Chem..

[6] Stephens C., Tanious F., Kim S., Wilson W., Schell W., Perfect J., Franzblau S., Boykin D. (2001). J. Med. Chem..

[7] Stephens C., Brun R., Salem M., Werbovetz K., Tanious F., Wilson W., Boykin D. (2003). Bioorg. Med. Chem. Lett..

[8] Reid C., Farahat A., Zhu X., Pandharkar T., Boykin D., Werbovetz K. (2012). Bioorg. Med. Chem. Lett..

[9] Rostovtsev V., Green L., Fokin V., Sharpless K. (2002). Angew. Chem., Int. Ed..

[10] Yuste F., Saldana M., Walls F. (1982). Tetrahedron Lett..

[11] van Leusen A., Hoogenboom B., Siderius H. (1972). Tetrahedron Lett..

[12] Hoarau C., Du Fou de Kerdaniel A., Bracq N., Grandclaudon P., Couture A., Marsais F. (2005). Tetrahedron Lett..

[13] Besselievre F., Mahuteau-Betzer F., Grierson D., Piguel S. (2008). J. Org. Chem..

[14] Delfín D.A., Morgan R.E., Zhu X., Werbovetz K.A. (2009). Bioorg. Med. Chem..

[15] Zhu X., Pandharkar T., Werbovetz K. (2012). Antimicrob. Agents Chemother..

[16] Zhu X., Van Horn K., Barber M., Yang S., Wang M.Z., Manetsch R., Werbovetz K. (2015). Bioorg. Med. Chem..

[17] Pandharkar T., Zhu X., Mathur R., Jiang J., Schmittgen T., Shaha C., Werbovetz K. (2014). Antimicrob. Agents Chemother..

[18] de Macedo-Silva S., Visbal G., Urbina J., de Souza W., Rodrigues J. (2015). Antimicrob. Agents Chemother..

[19] Paniz Mondolfi A., Stavropoulos C., Gelanew T., Loucas E., Perez Alvarez A., Benaim G., Polsky B., Schoenian G., Sordillo E. (2011). Antimicrob. Agents Chemother..

[20] Saenz R., Paz H., Berman J. (1990). Am. J. Med..

[21] Beach D., Goad L., Holz G. (1988). Mol. Biochem. Parasitol..

[22] Buckner F., Wilson A. (2005). Am. J. Trop. Med. Hyg..

[23] Peters W., Ekong R., Robinson B., Warhurst D., Pan X. (1990). Ann. Trop. Med. Parasitol..

[24] de Macedo-Silva S., Urbina J., de Souza W., Rodrigues J. (2013). PLoS One.

[25] Banerjee M., Farahat A., Kumar A., Wenzler T., Brun R., Munde M., Wilson W., Zhu X., Werbovetz K., Boykin D. (2012). Eur. J. Med. Chem..

