# Supplementary material for: Synthesis and pharmacological evaluation of mono-arylimidamides as antileishmanial agents
Source: Bioorg Med Chem Lett. 2016 May 15;26(10):2551–6. doi: 10.1016/j.bmcl.2016.03.082 (PMC4841789; doi:10.1016/j.bmcl.2016.03.082)
Supplement: Supplementary data — Complete characterizations of target compounds 1b, 1d, 1k, 2, and 3. [file mmc1.docx]

**Synthesis and Pharmacological Evaluation of Mono-arylimidamides as Antileishmanial Agents**

Xiaohua Zhu, Abdelbasset A. Farahat, Meena Mattamana, April Joice, Trupti Pandharkar, Elizabeth Holt, Moloy Banerjee, Jamie L. Gragg, Laixing Hu, Arvind Kumar, Sihyung Yang, Michael Zhuo Wang, David W. Boykin, and Karl A. Werbovetz^*^

**Supplementary information**

**Synthesis.** A general procedure for the preparation of the ﬁnal products **1b**, **1d**, **1k**, **2**, and **3** is given below as a representative method used for all final compounds; characterization data is also included. All solvents and reagents were used without puriﬁcation as acquired from commercial sources. Melting points were measured using a capillary melting point apparatus which was uncorrected. Progress of the chemical reactions was monitored by thin-layer chromatography on silica gel 60-F254 aluminum plates and detected under UV light. All NMR spectra were recorded employing a 400 MHz spectrometer, and chemical shifts (δ) are in ppm relative to TMS as internal standard. The ^13^C NMR signal for the mesylate methyl group is often overlapped by the methyl signal of the DMSO (solvent); measurements in methanol-*d_4_* are often free from this problem (data not shown). Electrospray ionization (ESI) Q-Tof and Orbitrap were used for the mass spectra measurements. Elemental analyses are within ±0.4 of the theoretical values. Compounds reported as salts frequently analyzed for fractional moles of water and other solvents (e. g. ethanol) of solvation. In each case proton NMR showed the presence of the indicated solvent(s).

**General Procedure for Synthesis of [2-Alkoxy-4-(2-pyridylimino and pyrimidylimino)aminophenyl]-arylheterocycles methanesulfonates or hydrochlorides.** *S*-(2-Naphthylmethyl)-2-pyridyl or pyrimidyl thioimidate hydrobromide (1.87 mmol) was added to a cooled solution of the appropriate aryl amine (1.7 mmol) in a mixture of dry ethanol (20 mL) and dry acetonitrile (10 mL) cooled in an ice bath. The reaction mixture was stirred at room temperature overnight. After the disappearance of the starting material (TLC), the organic solvent was evaporated under reduced pressure to yield a crude oily product. Dry ether (200 mL) was added to the crude material and the mixture was stirred at room temperature for 4 h. The precipitate was filtered and washed with dry ether. The solid was dissolved in ethanol (5 ml); the solution was cooled to 0 ^o^C in an ice bath and 10% NaOH was added until a pH of approximately 10 was reached. The free base was extracted with ethyl acetate (3 × 100 mL). The organic layer was washed with distilled water, dried over anhydrous K_2_CO_3_, filtered and concentrated under reduced pressure. Dry hexane was added to the resulting suspension and then filtered. The free base was suspended in dry dichloromethane (15 mL) and cooled to 0 ^o^C in an ice bath. Freshly distilled methanesulfonic acid (0.179 g, 1.87 mmol) was added to the suspension and the mixture was stirred at room temperature for 0.5 h. The resulting red solution was concentrated under reduced pressure. The crude red solid was crystallized from dry dichloromethane and dry ether and was then filtered. For the hydrochloride salts the free base was added to ethanolic HCl for 4 h, ether was added and the salt was obtained by filtration.

***N*-(3-isopropoxy-4-(5-phenylfuran-2-yl)phenyl)picolinimidamide methanesulfonate 1b.** Yellow solid, yield 65%; mp 177–178 ^o^C dec.; ^1^H NMR (400 MHz, DMSO-*d_6_*) δ 11.75 (s, 1H), 10.06 (s, 1H), 9.36 (s, 1H), 8.93 (d, 1H, J = 4.8 Hz), 8.38 (d, 1H, J = 8.0 Hz), 8.27-8.23 (m, 1H), 8.17 (d, 1H, J = 8.4 Hz), 7.89-7.85 (m, 3H), 7.50-7.46 (m, 2H), 7.59 (d, 2H, J = 8.0 Hz), 7.17-7.13 (m, 3H,), 4.85-4.81 (m, 1H), 2.32 (s, 3H), 1.33 (brs, 6H); ^13^C NMR (100 MHz, DMSO-*d_6_*) δ 160.0, 154.5, 152.4, 150.3, 149.2, 145.0, 138.9, 134.5, 130.4, 129.4, 129.0, 128.2, 126.9, 124.4, 124.0, 120.0, 118.2, 113.5, 111.7, 108.8, 71.8, 22.4, 15.6; HRMS (ESI) calcd for C_25_H_24_N_3_O_2_ (M^+^ + H) 398.1863, found 398.1851; Anal. Calcd. For C_25_H_23_N_3_O_2_ • CH_3_SO_3_H •0.5H_2_O: C, 62.13; H, 5.61; N, 8.36. Found: C, 62.10; H, 5.48; N, 8.24.

***N*-(4-(2,2’-bifuran-5-yl)-3-isopropoxyphenyl)picolinimidamide hydrochloride 1d.** Yellow solid, yield 43%; mp 195–197 °C dec. ^1^H NMR (400 MHz, DMSO-*d_6_*): δ 11.85 (s, 1H), 10.16 (s, 1H), 9.37 (s, 1H), 8.89 (d, *J* = 4.4 Hz, 1H), 8.52 (d, *J* = 7.6 Hz, 1H), 8.22 (t, *J* = 7.6 Hz, 1H), 8.00 (d, *J* = 8.0 Hz, 1H), 7.85 (t, *J* = 5.2 Hz, 1H), 7.79 (s, 1H), 7.31 (s, 1H), 7.15–7.11 (m, 2H), 6.85 (dd, *J* = 10.8 Hz, 3.2 Hz, 2H), 6.65 (s, 1H), 4.83–4.80 (m, 1H), 1.41 (d, *J* = 5.6 Hz, 6H); ^13^C NMR (100 MHz, DMSO-*d_6_*): δ 159.3, 153.9, 149.7, 148.5, 145.4, 144.6, 144.4, 143.1, 138.3, 134.2, 128.5, 126.2, 124.1, 118.9, 117.6, 112.4, 111.9, 111.2, 107.7, 106.1, 70.6, 21.8; HRMS (ESI) calcd for C_23_H_21_N_3_O_3_ (M^+^ + H) 388.1661, found 388.1648; Anal. Calcd for C_23_H_21_N_3_O_3_ •1.5HCl, H_2_O: C, 60.04; H, 5.37; N, 9.13. Found: C, 60.37; H, 5.15; N, 9.11.

***N*-(3-(cyclopentyloxy)-4-(5-phenylfuran-2-yl)phenyl) picolinimidamide methanesulfonate 1k.** Yellow solid, yield 59% ; mp < 200 ^o^C dec.; ^1^H NMR (400 MHz, DMSO-*d_6_*) δ 11.90 (s, 1H), 10.05 (s, 1H), 9.34 (s, 1H), 8.92 (brs, 1H), 8.39 (d, *J* = 7.2 Hz, 1H), 8.27 (t, *J* = 8 Hz, 1H), 8.13 (dd, *J* = 2.0, 8.0 Hz, 1H), 7.87-7.85 (m, 3H), 7.49-7.42 (m, 2H), 7.35 (t, *J* = 7.2 Hz, 1H), 7.29 (s, 1H), 7.17-7.12 (m, 2H), 7.08 (brs, 1H), 5.05 (brs, 1H), 2.34 (s, 3H), 2.01-1.69 (m, 4H), 1.80 (brs, 2H), 1.68 (brs, 2H); ^13^C NMR (100.0 MHz, DMSO-*d_6_*) δ 159.0, 154.1, 153.0, 150.3, 149.7, 145.9, 138.7, 134.5, 130.4, 129.4, 129.0, 128.2, 126.9, 124.4, 124.5, 120.3, 118.7, 113.9, 111.8, 109.1, 78.7, 32.7, 24.5; HRMS (ESI) calcd for C_27_H_26_N_3_O_2_ (M^+^ + H): 424.2020, found: 424.2010; Anal. Calcd. for C_27_H_25_N_3_O_2_ •CH_3_SO_3_H •1.15H_2_O: C, 62.24; H, 5.83; N, 7.77. Found: C, 62.41; H, 6.01; N, 7.69.

***N*-(3-isopropoxy-4-(1-(4-methoxyphenyl)-1*H*-1,2,3-triazol-4-yl)phenyl)picolinimidamide methanesulfonate 2.** Orange solid, yield 92%; mp 288–291 (dec). ^1^H NMR (400 MHz, DMSO-*d_6_*) δ 11.75 (brs, 1H), 10.08 (brs, 1H), 9.41 (brs, 1H), 8.92 (d, J = 4 Hz, 1H), 8.74 (s, 1H), 8.40 (d, J = 8.2 Hz, 1H), 8.34 (d, J = 8.2 Hz, 1H), 8.25 (t, J = 8 Hz, 1H), 7.87 (d, J = 8 Hz, 3H), 7.36 (s, 1H), 7.19 (d, J = 8 Hz, 3H), 4.83 (m,1H), 3.85 (s, 3H), 2.31 (s, 3H), 1.45 (d, J = 6 Hz, 6H) ^13^C NMR (100 MHz, DMSO-*d_6_*): δ (ppm): 159.5, 159.4, 154.5, 149.9, 144.6, 142.4, 138.4, 135.0, 130.0, 128.6, 128.3, 123.9, 122.2, 121.7, 119.5, 117.7, 115.0, 111.2, 70.9, 55.6, 21.8. HRMS (ESI) calcd for C_24_H_25_N_6_O_2_: 429.2034, found: 429.2023. Anal.: Calc. for C_24_ H_2~~4~~_N_6_O_2_•1.0CH_3_SO_3_H •1.0H_2_O: C, 55.37; H, 5.58; N, 15.50. Found: C, 55.41; H, 5.44; N, 15.24.

***N*-(3-isopropoxy-4-(2-phenyloxazol-5-yl)phenyl)picolinimidamide methanesulfonate 3.** Orange solid, yield 95%; mp 282–285 º(dec). ^1^H NMR (400 MHz, DMSO-*d_6_*) δ 11.75 (brs, 1H), 10.07 (brs, 1H), 9.39 (brs, 1H), 8.92 (d, J = 4.8 Hz, 1H), 8.38 (d, J = 8 Hz, 1H), 8.25 (t, J = 8 Hz, 1H), 8.10-8.15 (m, 3H), 7.87 (t, J = 4.8 Hz, 1H), 7.74 (s, 1H), 7.57-7.61 (m, 3H), 7.38 (s, 1H), 7.20 (d, J = 8 Hz, 1H), 4.87 (m, 1H), 2.30 (s, 3H), 1.44 (d, J = 6 Hz, 6H); ^13^C NMR (100 MHz, DMSO-*d_6_*) δ 159.6, 154.3, 149.8, 147.1, 144.5, 138.4, 135.1, 130.8, 129.2, 128.6, 127.7, 126.9, 126.7, 126.0, 123.8, 117.9, 116.8, 111.2, 107.2, 71.0, 21.8. HRMS (ESI) calcd for C_24_H_23_N_4_O_2_: 399.1816, found: 399.1806. Anal.: Calc. for: C_24_H_22_N_4_O_2_ •1.0CH_3_SO_3_H •1.2H_2_O: C, 58.17; H, 5.55; N, 10.85. Found: C, 58.17; H, 5.34; N, 10.70.
